# Current Challenges of Plastic Surgical Care in Sub-Saharan Africa (Maputo, Mozambique)

**DOI:** 10.1097/GOX.0000000000001893

**Published:** 2018-08-07

**Authors:** Kevin J. Guzman, Natacha Gemo, Deborah B. Martins, Pedro Santos, Daniel A. DeUgarte, Fatima Ademo, David Kulber, Celma Issufo

**Affiliations:** From the * Department of Surgery, David Geffen School of Medicine at UCLA, Los Angeles, Calif.; † Department of Surgery, Hospital Central de Maputo, Maputo, Mozambique; ‡ Department of Surgery, Matola Hospital, Matola, Mozambique; § Department of Surgery, Cedars Sinai Medical Center, Los Angeles, Calif.

## Abstract

**Background:**

Limited data exist on plastic surgery practices in Sub-Saharan Africa. The aim of this study was to characterize the spectrum of disease and operative procedures at a teaching hospital in Maputo, Mozambique to help understand the challenges of providing care for the local providers and to provide contextual relevance for training through partnerships.

**Methods:**

A mixed-methods approach was utilized to perform an ongoing needs assessment. A retrospective review was performed of plastic surgery operative records, ward admissions records, and death records in a tertiary-care hospital in Maputo, Mozambique for the period January 2015 to December 2015.

**Results:**

Limited resources (equipment, block-time, personnel, and perioperative services) were observed. The most common diagnoses for the 455 patients evaluated were burns (44%) and neoplasms (17%). Congenital abnormalities accounted for only 1% of the patient diagnoses. Of the 408 procedures performed, the majority were skin grafts (43%) and skin excisions (31%). Sepsis from burns accounted for 70% of documented deaths (14/20). The mean number of days to skin grafting for inpatients was 53 days.

**Conclusion:**

We observed a large burden of burns and skin graft procedures at a public referral teaching hospital in Mozambique. Our findings provide contextual relevance to help focus public health efforts and improve plastic surgery training and practices.

## INTRODUCTION

Recently, there has been increased interest in global surgery. Surgical disease in the developing world remains an unmet need, leading to significant morbidity and mortality.^1–4^ An estimated 11% of worldwide disability-adjusted life years are due to surgically treatable diseases, such as burns, trauma, and congenital anomalies.^5^ Plastic surgeons are uniquely equipped to handle many of these conditions, leading to lower rates of scar contractures, osteomyelitis, and improved outcomes for cleft lips/palates.^3^

Most studies in the plastic surgery literature describe mission trips that focus on highly specialized problems including congenital anomalies like cleft lip/palate. Little data exist on plastic surgery practices in sub-Saharan Africa and how best to improve care. Our organization has developed partnerships to promote training of local providers. Our goal was to characterize plastic surgery practices at Hospital Central Maputo (HCM) in Mozambique to help understand the challenges in delivering care for the local providers and to facilitate training.

## METHODS

Approval was obtained from the UCLA Institutional Review Board and from the Scientific Committee of HCM. To assess the resources and personnel, a mixed-methods approach was utilized that involved both quantitative and qualitative data collection, including key informant interviews with local and visiting physicians and surgeons, to perform an ongoing needs assessment of the plastic surgery service. Retrospective data were obtained from all patients admitted to the plastic surgery ward at HCM from January, 2015, to December, 2015. Patient age, sex, province, district, admission date, and discharge date were collected from the plastic surgery ward discharge logbook and the mortality logbook. Operative information (location of procedure, age, sex, province, and procedure performed) was obtained from plastic surgery inpatient and outpatient operative logbooks. Patient information was recorded onto a standardized form, which was later transferred into electronic records.

To evaluate the spectrum of diagnoses managed by the plastic surgery service, we combined discharge diagnosis data from admissions to the plastic surgery ward with primary diagnosis for all operations. In cases where the primary diagnosis listed was different between the operative logbook and the discharge logbook, the diagnosis from the operative logbook was used as it was considered to be more reliable. When determining the frequency of each diagnosis for the cohort, only one primary discharge diagnosis was used per patient admission. To evaluate the spectrum of operative procedures performed by the plastic surgery service, all operations were included. If multiple operations were performed on a single patient, each procedure was accounted for individually.

Pediatric age group was defined as age less than 15 years at the time of admission (per hospital protocol). Pediatric patients who underwent operations by the plastic surgery service but were admitted to wards other than the plastic surgery ward (eg pediatric ward or intensive care unit) had missing demographic data. However, their operative and diagnostic data were recorded and included in this study.

Demographic data were collected from the discharge and mortality logbooks of the plastic surgery ward. Overall, demographic data were missing for 8% (38/455) of patients. Statistical analysis was performed using chi-square analysis for categorical variables and *t* test for continuous variables.

## RESULTS

### Resources and Personnel

Hospital Central de Maputo is a 1,500-bed teaching hospital with an average daily census that can expand to over 3,000 during the rainy season. Plastic surgery is a division in the Department of Surgery, which also oversees general surgery, thoracic and vascular surgery, neurosurgery, urology, ophthalmology, otolaryngology, oromaxillofacial, and pediatric surgery. The plastic surgery ward has 6 rooms, of which 2 have 9 beds and 4 have 6 beds (total of 42 beds). There are no curtains or walls separating beds. There is no hospital infection control. Relatives are allowed to stay with a patient, and they are often allocated a chair to sit on; they often spend the night and, not infrequently, sleep in the same bed as the patient. Limited faucets, soap, and alcohol dispensers make hand hygiene challenging. Furthermore, the plastic surgery ward is not air conditioned or ventilated and temperatures can reach over 100 degrees Fahrenheit. There are limited supplies and pain-management resources (eg narcotics, sedation) to adequately perform daily wound care. Disposable and sterile gloves are limited; staff must prioritize patients for daily dressing changes. For patients with prolonged periods of immobilization, there are limited physical therapy resources to help avoid contractures. Additionally, there are limited antibiotics, albumin, blood products, and microbiology services (cultures and sensitivities). Furthermore, there is inadequate food and dietary services to optimize patient nutritional status.

There is a dedicated procedure room in the plastic surgery wing for small procedures. Examples of these procedures include removal of stitches, minor wound debridement, wound dressing change, and corticosteroid injection. This room does not have the equipment necessary for conscious sedation. All procedures requiring sedation are performed in a separate building, where the operating rooms are located.

Patients often have delayed first operations as there is limited operating time. Patients presenting with burns are often managed conservatively with wound dressing and silver sulfadiazine, sodium hypochlorite, normal saline, or Vaseline gauze depending on the severity of the wound and availability of products. Acetic acid is reserved for wounds for which *P. aeruginosa* is suspected. The operating wing is equipped with 10 operating rooms, some of which have multiple operating beds to maximize space available for surgeries. Operating rooms are shared by multiple surgery departments. The plastic surgery team has 2 block days per week. Emergency plastic surgery operations can be performed on a case-by-case basis in a separate operating room facility near the emergency room.

Limited equipment is available for plastic surgery procedures. Only 2 electrical dermatomes and 4 meshers (with no new plates) are available to harvest skin for skin grafts. These devices are only used once per day as sterilization is only performed overnight. Once all dermatomes have been used for the day, knives are used to harvest skin. Patients who undergo surgeries are generally admitted to the plastic surgery ward. Some patients may be transferred to the intensive care unit or pediatric ward.

Currently, there are 3 plastic surgeons at HCM, 2 of which are Mozambican, 1 of whom serves as Chief of Plastic Surgery. In total, Mozambique has 3 Mozambican plastic surgeons and 3 Cuban plastic surgeons. Two surgeons are located in the north and central regions of the country. The remaining 4 surgeons are located in Maputo province, where the capital city is located. All plastic surgeons also work in the private sector to supplement their incomes.

### Plastic Surgery Patients—Demographic Information

A total of 455 patients were either admitted to the plastic surgery ward or underwent an operation. The majority of patients were from the southern provinces of Mozambique (n = 393), of which 71% (n = 325) were from the Maputo City or Maputo province. These are the 2 most populous cities of the country and the nearest to HCM. The mean age at admission was 29 years. Patients managed nonoperatively represented 32% (145/455) of all patients. Of those patients who were admitted to the plastic surgery ward, patients managed operatively had a significantly longer length of stay than those managed nonoperatively (55 versus 25 d, *P* < 0.001).

### Plastic Surgery Ward Discharge Diagnosis (Table 1) and Demographics

Overall, burns and burn complications were the most frequent diagnoses among all patients. Of all patients seen, 44% (n = 201) had either burns or burn complications. Neoplastic disease was the second most frequent diagnosis, followed by scar treatment, infections, ulcers, cyst, cosmetic treatment, and trauma (Table 1).

**Table 1.**
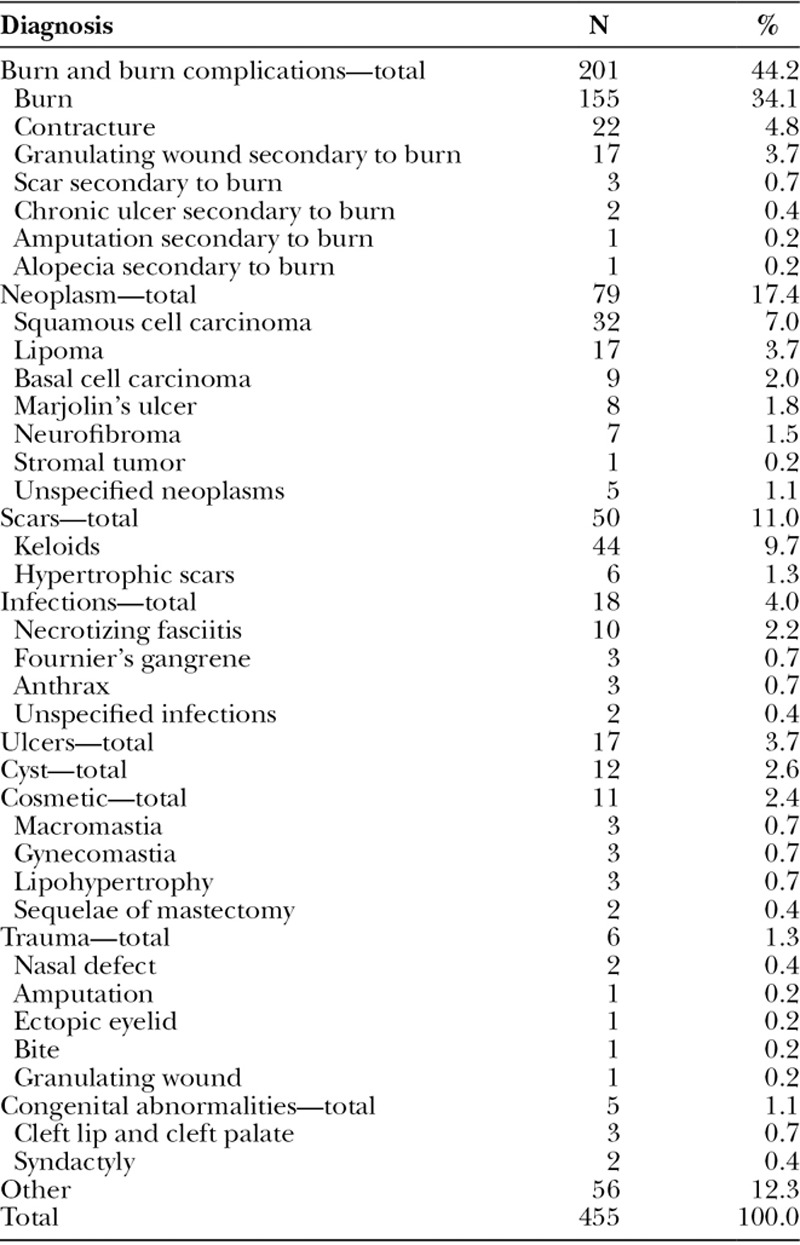
Diagnostic Categories for Plastic Surgery Ward and Operative Patients

**Fig. 1.**
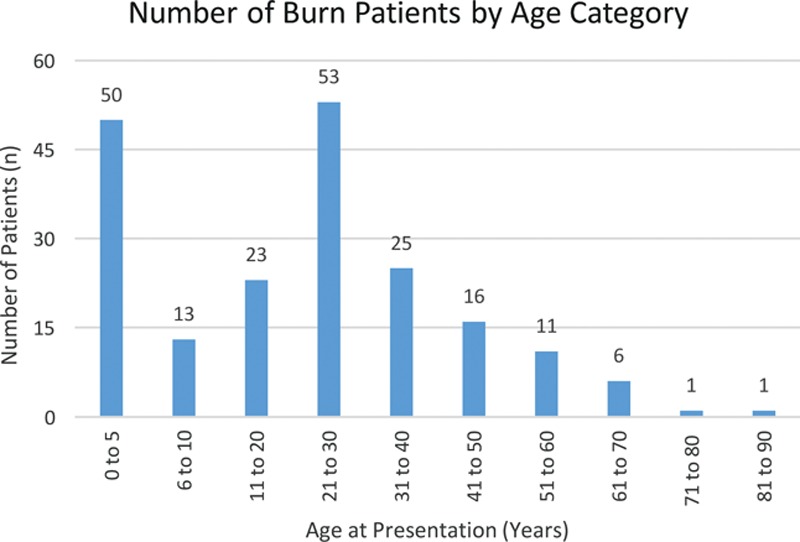
Distribution of age for burn patients.

Patients in this burns category include those with acute burns (n = 155), contractures (n = 22), granulating wounds (n = 17), scars (n = 3), and ulcers (n = 2) as a result of burns. Most burn patients were male (65%). The mean age was lower than that for other diagnoses treated by plastic surgery (24 versus 29 y; *P* < 0.01). A bimodal age distribution was observed with peaks at less than 5 years of age and between 21 and 30 years of age (Fig. 1). Burns were most likely to be seen in the upper limbs (n = 71), followed by the lower limbs (n = 38), and trunk (n = 33). Compared to patients with other diagnoses, burn patients were more likely to be treated nonoperatively (55% versus 89%; *P* < 0.001); they accounted for 78% of all patients treated nonoperatively. Mean hospital stay for patient undergoing an operation for burns was 37 days. These patients primarily received skin grafts (89/138).

Neoplastic disease was the second leading diagnosis seen on the plastic surgery ward, accounting for 17% (n = 79) of all patients seen. The majority of patients were female (64%). Patients with neoplastic disease were significantly older than patients with other diagnoses (38 versus 29 y; *P* < 0.001). Patients presented with squamous cell carcinoma (n = 32), lipomas (n = 17), basal cell carcinoma (n = 9), Marjolin’s ulcer (n = 8), and neurofibromas (n = 7). Almost all patients in this category of admissions were treated operatively (92%), of which excision of neoplasm was the most common procedure performed (n = 69). Patients were treated both in an inpatient (n = 41) and outpatient setting (n = 37). Neoplasms were most likely to be seen in the head (n = 47). Other common sites were the trunk (n = 13) and upper limb (n = 10).

Scar disease was another common diagnosis seen in the plastic surgery ward (11.0%, n = 50), of which keloids were the most likely diagnosis (n = 44). Most patients were female (58%). Patient with scar disease were more likely to be treated with outpatient surgery when compared with those with other diagnoses (78% versus 21%, *P* < 0.01). Most of these patients (96%) were treated operatively with excisions (n = 4 4). Keloids were especially common in the earlobes, and the majority of patients had scar disease of the head (n = 37).

The fourth (n = 18, 4.0%) leading diagnosis was of infectious disease etiology. The majority of patients were female (55%) and from the Maputo province (65%). Patients in this group were significantly older than those with other diagnoses (41 versus 29 y; *P* < 0.001). Infections were mostly seen in the lower limb (65%, n = 11). The most common infections seen were necrotizing fasciitis (n = 10), Fournier’s gangrene (n = 3), and anthrax (n = 3). Most patients were treated as inpatients (90%) and underwent an operation (80%) with skin grafts (n = 13).

Congenital defects were a small proportion (1.1%, n = 5) of diagnosis in the plastic surgery ward. Most patients were female (n = 4). All were treated operatively. Diagnoses in this category include cleft lip/palate (n = 3) and syndactyly (n = 2).

### Plastic Surgery Procedures Performed (Table 2)

Overall, 408 operations were performed on 310 patients. The majority were treated on an inpatient basis (n = 216, 70%). Most patients came from Maputo province (67%) and were female (55%). Mean age was 28 years. Skin grafts, excisions of lesions, reconstructions, contracture releases, and flaps were common operations performed.

**Table 2.**
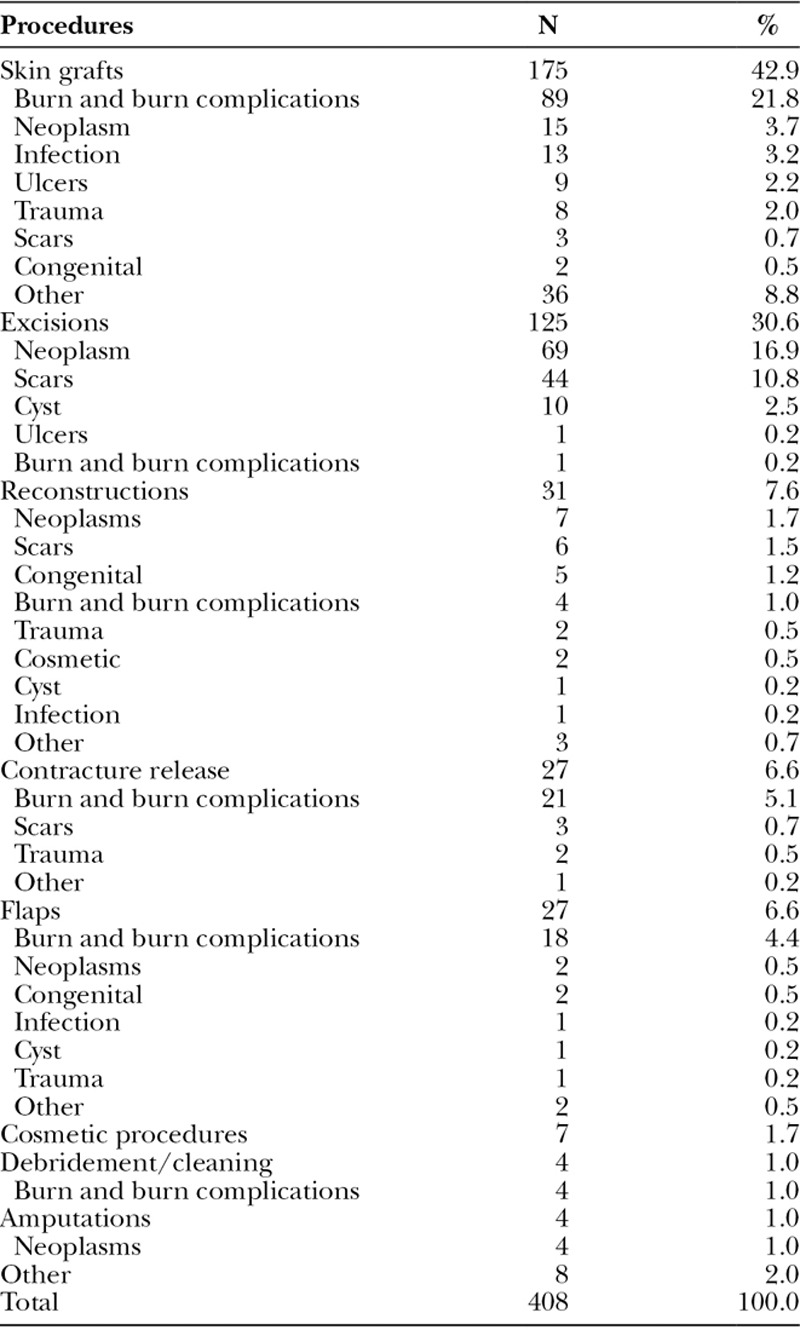
Operations Performed by the Plastic Surgery Service Subcategorized by Diagnostic Category

Skin grafts were the most common operation performed (n = 175, 43%). The majority (n = 89, 51%) of skin grafts were performed for patients with burns (n = 53) or burn complications (n = 46). Neoplasms were the second most common indication for a skin graft (n = 15), followed by infections (n = 13), ulcers (n = 9), traumatic disease (n = 8), and scars (n = 3).

**Fig. 2.**
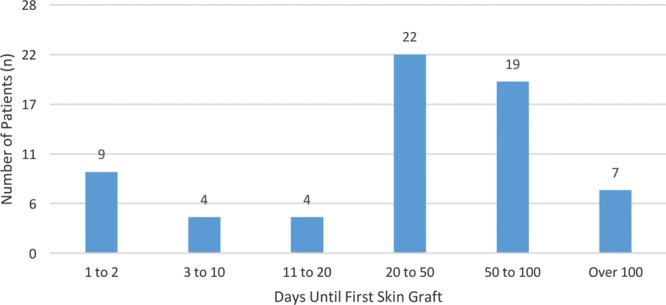
Time (days) until first skin graft. The mean days to skin graft for inpatients were 20 days. Only 9 received a skin graft within 1–2 days of arrival.

Of the burn patients who were treated operatively (n = 90), the majority of patients received a skin graft (n = 80, 89%). Mean number of days to skin graft was 53 days (Fig. 2). Burn patients receiving skin grafts had a significantly longer length of stay than burn patients without skin grafts (70 versus 37 d; *P* < 0.001). The majority of patients undergoing skin grafting were pediatric patients <15 years of age (51%), of which 80% were under age 5.

Excisions of lesions were the second most common (n = 125, 31%) operation performed. Excisions were primarily performed on patients presenting with neoplastic disease (n = 69, 55%). Seventy-one percent of operative patients presenting with neoplastic disease were treated by excision. Squamous cell carcinomas (n = 28) and lipomas (n = 17) were the most common diseases associated with excisions. Patients presenting with scars, mainly keloids, were also treated by excision (73%). Scars accounted for 35% (n = 44) of all excision procedures. Cyst (n = 10), burn complications (n = 1), and ulcers (n = 1) were also occasionally treated with excision.

Reconstructive procedures were the third most commonly performed procedure (n = 31, 8%). Procedures were performed for a variety of reasons including neoplastic disease (n = 7), scars (n = 6), congenital anomalies (n = 5), and burn and burn complications (n = 4). Cleft lip was repaired with a modified Millard procedure (n = 3).

Contracture release was less commonly performed (n = 27, 7%). Most of the patients who underwent contracture release had a diagnosis of burn complication (n = 21, 72%). Patients presenting with contractures due to scars (n = 3) and trauma (n = 1) also had contracture releases.

Flaps (n = 27, 7%) were primarily performed for patients presenting with burns and burn complications (n = 18, 67%). This was a common procedure performed on patients presenting with burn complications (n = 14). Neoplastic disease (n = 2), congenital anomalies (n = 2), infections (n = 1), cysts (n = 1), and traumatic disease (n = 1) were also indications for flap procedures. For the 19 patients for whom the flap type was documented, the distribution was as follows: Z-plasty (n = 8), advancing flaps of unspecific tissue (n = 6), and adipocutaneous flaps (n = 5).

Marjolin’s ulcers were the primary indication for amputation (n = 3, 75%). Two of these amputations were supracondylar and 1 was hemi-humeral. There was also a trans-tibial amputation of the leg for squamous cell carcinoma (n = 1).

Other procedures included mastectomies (n = 3), reduction mammoplasty (n = 3), liposuction (n = 1), and unspecified (n = 8).

### Mortality

Mortality was documented in the logbook for 20 of the 361 patients admitted to the plastic surgery ward (6%). Sepsis was the primary reason for death (n = 17, 80%). The diagnoses associated with sepsis included burns (n = 14), necrotizing fasciitis (n = 1), Marjolin’s ulcer complicated by pressure ulcer (n = 1), and nonspecific ulcer (n = 1). Three of the patient deaths had poor documentation for the cause of death. The majority of patients with documented death did not undergo an operation (90%). They were also significantly older (40 versus 28 y, *P* < 0.01). Patients were in the hospital for an average of 38 days before death.

## DISCUSSION

This study characterizes the spectrum of diagnoses and operative case volume of a plastic surgery division in a referral teaching hospital in sub-Saharan Africa country. Goodacre^6^ estimated that 16% of Tanzania’s surgical cases in a rural hospital would fall under the care of plastic surgeons. Our data demonstrate a large burden of burns in the plastic surgery ward, which is consistent with other data on surgical disease of sub-Saharan Africa.^7^ Furthermore, burns accounted for 19.1% of children seen in the emergency department in Mozambique.^11^ Like previous studies, we observed that toddlers (children under 5 y) are particularly susceptible to burns.^8,9^ Previous research has found scald water burns in addition to open fire cooking and crowded home conditions contribute to burns in Mozambique.^10–12^ We also identified a second peak of burns in patients aged 21 to 30. Further research is needed to elucidate the cause of burn in young adults.

Of note, burn management is critical to prevent progression to greater disability and suffering.^13^ In contrast to early closure practices in the United States, delayed closure (>5 d) has been shown to lead to better survival in burn patients in sub-Saharan Africa.^15,16^ Nonetheless, we noted a mean day to skin grafting of 53 days, which may contribute to higher rates of wound infection and contracture. Several factors may contribute to extreme delays in skin grafting (>100 d) including limited operative time, sepsis, and delays in medical optimization of the patient. Additional study is required on how best to expedite definitive treatment and improve outcomes.

Our study also describes some of the challenges in patient care and operative management. Limitations in operative block time, equipment (eg dermatomes and meshers), and sterilization procedures impede the ability to manage a high volume of burn patients. Perioperative care is also limited by a shortage of nurses and physical therapists, wound-care supplies, and pain management to adequately perform daily wound care. Patients often develop contractures likely because of periods of prolonged immobilization. In addition, there are limited resources for infection control (eg sinks for handwashing; alcohol-sanitizer dispensers; wound-care supplies; isolation beds; microbiology culture/sensitivity to guide therapy). Our findings provide a background that can be used to identify areas of future assistance, such as improving resources and facilities. The development of a dedicated burn unit would be beneficial in this regard.^14^

There are several limitations to the study. Notably absent from the case logs are craniofacial, microsurgery, hand, and cosmetic cases. Other surgical services care for patients with cleft lip/palate at HCM (ie oral maxillofacial surgery). Microsurgical cases are not performed because of the lack of operative microscopes and adequate postoperative care. Hand cases are managed by the orthopedic service. Cosmetic cases were not captured in this data set because they are managed in a separate private clinic. We were unable to capture data for minor procedures performed without sedation in the procedure room of the plastic surgery ward because of lack of documentation; however, these cases are uncommon. Furthermore, we were unable to capture data on outpatient and inpatient consultations on patients who did not undergo an operation. In addition, approximately 8% of operative patients did not have demographic or mortality data collected because they were admitted to a ward other than plastic surgery. Some of these patients were admitted to an intensive care ward, where the mortality rate may be higher. Therefore, the mortality rate we report is likely an underestimate. Another contributor to mortality that we do not address is coinfection with HIV, which has a high prevalence in HCM. Overall, the 6% mortality rate is high with the most common cause of death being sepsis from burn complications. Finally, a limitation of this study is that our findings at a single institution may not be generalizable across the region.

Our findings provide contextual relevance to help focus public health efforts and improve plastic surgery training and practices in similar settings. As a consequence, our partnership has chosen not to focus on specific disease or conditions (eg cleft lip/palate). Instead, it has focused its efforts on training in perioperative care and improving the skills of local surgeons. Our annual short-term intensive surgical missions include a team of nurses, wound-care specialists, anesthesiologists, and surgeons, who integrate with local providers into the daily care provided by the plastic surgery division. The mentorship and proctoring have extended beyond the period of the surgical mission, providing local surgeons with short-term apprenticeships abroad and immediate access for phone/video consultation. In 2017, 2 native plastic surgeons became fellows of the College of Surgeons of East, Central and Southern Africa (COSECSA). In summary, this study and its characterization of resources, spectrum of disease, and plastic surgery practices provide a framework to better understand the local needs and facilitate capacity-building efforts.

## ACKNOWLEDGMENTS

This research has been supported in part by the UCLA Center for World Health, Mending Kids International, and the NIH/NCRR/NCATS UCLA CTSI Grant UL1TR000124. We acknowledge the support of leadership of the Universidade Eduardo Mondlane medical school including Drs. Mamudo Ismael and Mohsin Sidhat, and leadership support of the Hospital Central Maputo, including Drs. Joao Fumane and Sandra Mavale. Funding sources were not involved in the study design, data analysis, writing, or submission of this article. The findings and conclusions presented are those of the authors and do not necessarily represent the official position of the funding agencies. We would also like to thank Dr. Thomas Coates, Director of the UCLA Center for World Health, Lee Miller, Director of the Global Health Education Program, and Dr. W. Chris Buck, Director, Mozambique Partnership for the UCLA Center for World Health.

## References

[1] Kruk ME Wladis A Mbembati N Human resource and funding constraints for essential surgery in district hospitals in Africa: a retrospective cross-sectional survey. PLoS Med. 2013;7:1 11. 10.1371/journal.pmed.1000242 PMC2834706 20231869

[2] Grimes CE Law RS Borgstein ES Systematic review of met and unmet need of surgical disease in rural sub-Saharan Africa. World J Surg. 2012;36:8 23. 22057752 10.1007/s00268-011-1330-1

[3] Semer NB Sullivan SR Meara JG Plastic surgery and global health: how plastic surgery impacts the global burden of surgical disease. J Plas Reconstr Aesthet Surg. 2010;63:1244 1248. 10.1016/j.bjps.2009.07.028 19700380

[4] Corlew DS Perspectives on plastic surgery and global health. Ann Plast Surg. 2009;62:473 477. 19387143 10.1097/SAP.0b013e31818c4b58

[5] Debas HT Gosselin R McCord C Chapter 67 Surgery. In: Disease Control Priorities in Developing Countries. 2006:2nd ed New York: Oxford University Press; 1245e60.

[6] Goodacre TE Plastic surgery in a rural African hospital: spectrum and implications. Ann R Coll Surg Engl. 1986;68:42 44. 3947014 PMC2498177

[7] Jovic G Corlew DS Bowman KG Plastic and reconstructive surgery in Zambia: epidemiology of 16 years of practice. World J Surg. 2012;36:241 246. 21725696 10.1007/s00268-011-1158-8

[8] Odeyinde SO Ademola SA Oluwatosin OM Predictors of mortality in paediatric burn at Ibadan, Nigeria. Afr J Paediatr Surg. 2007;4:29 32.

[9] Van Niekerk A Rode H Laflamme L Incidence and patterns of childhood burn injuries in the Western Cape, South Africa. Burns. 2004;30:341 347. 15145192 10.1016/j.burns.2003.12.014

[10] Karan A Amado V Vitorino P Evaluating the socioeconomic and cultural factors associated with pediatric burn injuries in Maputo, Mozambique. Pediatr Surg Int. 2015;31:1035 1040. 26280740 10.1007/s00383-015-3761-5 PMC4609601

[11] de Sousa Petersburgo D Keyes CE Wright DW The epidemiology of childhood injury in Maputo, Mozambique. Int J Emerg Med. 2010;3:157 163. 21031039 10.1007/s12245-010-0182-z PMC2926875

[12] Wesson HK Bachani AM Mtambeka P Pediatric burn injuries in South Africa: a 15-year analysis of hospital data. Injury. 2013;44:1477 1482. 23415388 10.1016/j.injury.2012.12.017

[13] Mock C Peck M Krug E Confronting the global burden of burns: a WHO plan and a challenge. Burns. 2009;35:615 617. 19423230 10.1016/j.burns.2008.08.016

[14] Al-mousawi AM Mecott-rivera GA Jeschke MG Burn teams and burn centers: the importance of a comprehensive team approach to burn care. Clin Plast Surg. 2009;36:547 554. 19793550 10.1016/j.cps.2009.05.015 PMC2801053

[15] Prasanna M Mishra P Thomas C Delayed primary closure of the burn wounds. Burns. 2004;30:169 175. 15019128 10.1016/j.burns.2003.09.028

[16] Gelaye B Rondon M Araya PR Timing of early excision and grafting following burn in Sub-Saharan Africa. Burns. 2015;4:1353 1359. 10.1016/j.burns.2015.02.011 PMC5171218 26088149

